# Integrating data from randomized controlled trials and observational studies to predict the response to pregabalin in patients with painful diabetic peripheral neuropathy

**DOI:** 10.1186/s12874-017-0389-2

**Published:** 2017-07-20

**Authors:** Joe Alexander, Roger A. Edwards, Alberto Savoldelli, Luigi Manca, Roberto Grugni, Birol Emir, Ed Whalen, Stephen Watt, Marina Brodsky, Bruce Parsons

**Affiliations:** 1 Pfizer Inc, 235 E 42nd St, New York, NY 10017 USA; 2 Health Services Consulting Corporation, 169 Summer Road, Boxborough, MA 01719 USA; 3 Fair Dynamics Consulting, srl, Via Carlo Farini, 5, 20154 Milan, Italy; 4 Pfizer Inc, Eastern Point Rd, Groton, CT 06340 USA

**Keywords:** Coarsened exact matching, Diabetic peripheral neuropathy, Neuropathic pain, Autoregressive models, Covariate bias, Pregabalin, Sleep interference, Hierarchical cluster analysis

## Abstract

**Background:**

More patient-specific medical care is expected as more is learned about variations in patient responses to medical treatments. Analytical tools enable insights by linking treatment responses from different types of studies, such as randomized controlled trials (RCTs) and observational studies. Given the importance of evidence from both types of studies, our goal was to integrate these types of data into a single predictive platform to help predict response to pregabalin in individual patients with painful diabetic peripheral neuropathy (pDPN).

**Methods:**

We utilized three pivotal RCTs of pregabalin (398 North American patients) and the largest observational study of pregabalin (3159 German patients). We implemented a hierarchical cluster analysis to identify patient clusters in the Observational Study to which RCT patients could be matched using the coarsened exact matching (CEM) technique, thereby creating a matched dataset. We then developed autoregressive moving average models (ARMAXs) to estimate weekly pain scores for pregabalin-treated patients in each cluster in the matched dataset using the maximum likelihood method. Finally, we validated ARMAX models using Observational Study patients who had not matched with RCT patients, using *t* tests between observed and predicted pain scores.

**Results:**

Cluster analysis yielded six clusters (287–777 patients each) with the following clustering variables: gender, age, pDPN duration, body mass index, depression history, pregabalin monotherapy, prior gabapentin use, baseline pain score, and baseline sleep interference. CEM yielded 1528 unique patients in the matched dataset. The reduction in global imbalance scores for the clusters after adding the RCT patients (ranging from 6 to 63% depending on the cluster) demonstrated that the process reduced the bias of covariates in five of the six clusters. ARMAX models of pain score performed well (*R* ^*2*^: 0.85–0.91; root mean square errors: 0.53–0.57). *t* tests did not show differences between observed and predicted pain scores in the 1955 patients who had not matched with RCT patients.

**Conclusion:**

The combination of cluster analyses, CEM, and ARMAX modeling enabled strong predictive capabilities with respect to pain scores. Integrating RCT and Observational Study data using CEM enabled effective use of Observational Study data to predict patient responses.

**Electronic supplementary material:**

The online version of this article (doi:10.1186/s12874-017-0389-2) contains supplementary material, which is available to authorized users.

## Background

Multiple interacting risk factors and comorbidities make it difficult to select the right treatment for the right patient experiencing neuropathic pain, including those with painful diabetic peripheral neuropathy (pDPN). pDPN presents in up to 26% of patients with diabetes mellitus [1], with age, duration of diabetes, and poor glycemic control as major factors in its development [2]. With the global prevalence of diabetes at 8.5% in 2014 [3] and the US prevalence at 9.3% [4], pDPN is a notable burden in over 2% of the global population. Neuropathic pain has a large variety of etiologies and many patients do not receive appropriate treatment for their pain [5], including those with pDPN. Reasons include shortfalls in proper patient and pain assessment, insufficient diagnostic accuracy, and inadequate knowledge about medications and their appropriate clinical use, combined with relatively limited treatment efficacy [5]. Patient, clinician, and health care system factors interact to affect these outcomes in pain [6–10].

From the COmbination versus Monotherapy of pregaBalin and dulOxetine in Diabetic Neuropathy Study (COMBO-DN) study, Bouhassira et al. [11] analyzed neuropathic pain sensory phenotypes in patients with painful diabetic neuropathy. They confirmed the advantages of sensory phenotypes and their predictive value, and thus concluded that heterogeneity of the patient populations should be taken into account for delivering more customized treatment. These results are consistent with both Freeman et al. [12] in terms of identifying clusters with distinct pain characteristics independent of neuropathic pain syndrome and with Baron et al. [13] in terms of pain-related sensory abnormality-based profiles as a way of identifying patient subgroups for treatment.

‘Omics’ and other emerging biomarker data combined with computational tools for exploring large datasets suggest how much more patient information can be utilized to deliver more customized care in general [14] and in neuropathic pain in particular [15]. Ongoing efforts strive to identify psychosocial variables that could be used to identify patient subgroups [16] as well, even if the end goal of fully personalized or precision medicine cannot be achieved in the short term [5]. Patient-centered care demands improved alignment of patient clinical needs with specific treatment strategies. These needs apply to patients with pDPN because of the demonstrated variability of response [17] and the devastating impact of insufficient pain relief (e.g., suffering, reduced physical activity, resultant increase in the risk of obesity with worsening of diabetes, comorbid cardiovascular conditions).

Significant resources are being invested to address this variation in patients’ responses to medical treatments more effectively [14] to meet expectations for more patient-specific care for chronic pain [14]. Health care is amidst a major transformation regarding how the overwhelming amount of patient data has become available via electronic health records and biomarkers, as well as how healthcare providers and patients may take advantage of social network and media data [18]. Such data are being used to help achieve ‘Triple Aim’ goals [19] of improving the health of populations, improving the patient experience of care, and reducing the per capita cost of care [20].

Realization of the clinical application of these enormous amounts of data will depend on the blending of evidence-based medicine from traditional clinical study sources together with ‘big data’ methods [18]. Addition of classification, data mining, and predictive analytic techniques have already enabled insights [14, 18], and additional efforts are required, such as those that can better link observational data with randomized data. Cameron et al. (2015) reviewed the advantages, disadvantages, and methodological challenges of linking the two types of studies in network meta-analyses and emphasized the importance of such efforts in generating evidence from across a medication’s lifecycle given the growth in analyses of post-approval data that needs to be combined with pre-approval data [21]. While traditional statistical techniques such as meta-analyses and network meta-analyses have supported linkage of different studies, they still generate population-level results, which require the clinician to further extrapolate them to individual patient treatment decisions. Improved methodological techniques for connecting data at the patient level are being developed (e.g., Iacus et al. (2012) on Coarsened Exact Matching (CEM) [22]) and provide better ways of integrating data from observational studies and RCTs. This goal of integrating RCT and observational study data guided our effort, and we started with the specific case of pain response in pDPN to treatment with the α2δ ligand, pregabalin, to demonstrate a proof of concept as to how such data integration could be implemented to improve outcomes. Understanding which patients are going to have a better-than-average response to treatment may shed light on the possible improvements in care that could increase the proportion of good responders. Efforts have evolved during the past two decades to predict individual patient responses via predictive analytics and simulation building on the pioneering work of David Eddy (2012) [23].

We sought to utilize a variety of these predictive analytics and simulation methods to link RCT data with Observational Study real-world data to predict responses to pregabalin in patients with pDPN. Pregabalin is approved in the United States for pDPN, among other uses [24]. Updated recommendations of the Special Interest Group on Neuropathic Pain (NeuPSIG) of the International Association for the Study of Pain included pregabalin, among other medications, as having a ‘strong’ GRADE recommendation as first-line therapy for neuropathic pain [5]. Systematic reviews and meta-analyses have noted that pDPN patient responses to pregabalin can vary [25–28]; less is understood about subgroups of patients in the studies who are most likely to respond. Our goal was to identify profiles of patients that reflect integrated RCT and Observational Study data to help clinicians treat pDPN more effectively by bridging the two types of evidence in a single platform to predict the potential level of response to pregabalin. The focus of the work described in this article is the generation of patient profiles based upon integration of RCT and Observational Study data; a follow-up article will demonstrate how such profiles can be utilized in a modeling and simulation environment to predict the probability of individual patients’ responses to drug therapy over time.

## Methods

We sought to use the RCT data to reduce the level of bias in the covariates’ distributions in the Observational Study data. A high degree of imbalance occurs more often in observational studies, which do not have random assignment to treatments. The reduction of imbalance can consequently occur when matching observational studies with RCTs in which the covariates are, in principle, more highly balanced due to the randomized design. Matching is intended to identify a better balance in the multidimensional distribution of covariates. Through the matching process, the matched data results in lower covariate bias and therefore establishes a basis for more explanatory models of potential causal relationships among measured variables [29]. We used CEM to match the RCT data to the Observational Study data [22]. We chose CEM because it is more precise than the often-used propensity score matching (PSM) approach and has a lower root mean square error [30, 31]. The other advantage is that CEM fixes imbalance ex ante and attempts to discard as few observations as possible ex post. This is in contrast to PSM, which fixes the matched sample size ex ante and attempts to reduce imbalance as a result of the procedure. This difference means that PSM discards considerable information ex ante and this PSM inefficiency can be considered a bias [32]. Moreover, CEM is superior to exact matching (EM) techniques. CEM overcomes the problem of limited numbers of matches, which happens quite often when EM techniques are applied due to the richness of the covariates in many cases [33]. In contrast to EM, which simply matches a treated unit to all the control units with the same covariate values, CEM relaxes these constraints by introducing classes of the covariates values to be matched. This matching reduces bias by decreasing the degree of dependence of the outcome variable on the estimation model [29].

For this proof of concept to link RCT data with Observational Study data, we began with the three pivotal studies for pregabalin, all of which contained the following data for patients receiving active treatment: age, gender, body mass index (BMI), baseline pain score (0–10 scale, with higher values indicating greater severity), baseline sleep interference score (0–10 scale, with higher values indicating more sleep disturbance), glycated hemoglobin (HbA1c) normal or elevated, insulin use, fixed doses of pregabalin monotherapy, duration of diabetes, allodynia at baseline, average weekly pain (based on daily scores), and average weekly sleep interference (based on daily scores). These studies were conducted in North America and described in prior publications [34–36]. For all studies, participants provided written informed consent, and all related study protocols were approved by the Institutional Review Boards and Ethics Committees of the investigators.

We also utilized the largest Observational Study of pregabalin, which contained the following data that overlapped with the RCT data: age, gender, BMI, baseline pain score, baseline sleep interference score, HbA1c (normal or elevated), and insulin use (yes or no). In contrast to the RCT dataset, the Observational Study did not have duration of diabetes and allodynia at baseline, but it did have flexible dosing of pregabalin monotherapy; duration of pDPN; prior gabapentin use; prior or current (at baseline) medical history of depression, sleep disorder , or anxiety; and general feeling responses to three questions on a six-point always-to-never scale (calm and relaxed, full of energy, discouraged) recorded at baseline and at Weeks 1, 3, and 6. To estimate missing data at Weeks 2, 4 and 5, we used the EXPAND SAS procedure that makes second-order interpolation. The Observational Study also had pain and sleep interference scores at baseline and at Weeks 1, 3, and 6 (in contrast to daily diary scores in the RCTs). This study was conducted in Germany and has been described in prior publications [37].

The first step before matching the two types of data was to better understand the characteristics of subgroups of patients in each dataset. We initiated our efforts with a hierarchical cluster analysis to identify ways patients might be grouped. Cluster analysis can be used to detect the presence of subpopulations within a dataset based on common statistical patterns. Given the variation in patients with pDPN described above, we thought the clustering would provide a useful approach. Clustering is also one way of reducing the chances of occurrence of Simpson’s paradox, in which a subgroup relationship differs from an overall population relationship [38]. Cluster analysis assigns individuals to groups (‘clusters’) who share certain similarities, in contrast with factor analysis, which uses inter-correlations among variables to form a smaller number of factors [16]. We chose Ward’s minimum variance technique, because it is considered one of the best methods for accuracy [39], and it also offers several additional advantages such as useful visualizations (dendrograms) that also guide in the selection of the cutoff point to determine the number of clusters. It also is a deterministic technique, thereby enabling results that are reliably reproducible [40]. We implemented this hierarchical cluster analysis first for patients in the three RCTs alone and then for patients in the Observational Study alone so as to better understand independently how patients from each of the two types of data were clustering before we matched RCT patients with Observational Study patients. After completing the clustering, we matched the RCT patients to the clusters identified in the Observational Study dataset. We chose this approach rather than starting with the RCT clusters because the observational study dataset was larger. The goal was to maximize the use of RCT patients and reduce the bias within each cluster with CEM [22].

This RCT–Observational Study matched dataset with the lower covariate bias achieved with CEM was then used for predicting responders. To that end, we implemented AutoRegressive Moving Average models with eXogenous inputs (ARMAX) to better represent multivariate time series analysis of the pain score at a given time lag in relation to: pain score at antecedent time lags (autoregressive part of the model); sleep interference score and other relevant time-dependent variables (e.g., general feeling variables) at different time lags (moving average part of the model); and specific patient demographic and/or medical history data likely to influence pain score (the exogenous inputs). ARMAX models are mathematical models of persistence, or autocorrelation, in a time series. They are used widely to predict the behavior of a time series from past values alone. Such a prediction can be used as a baseline to evaluate the possible importance of other variables to the system under study. We also used cross-correlation analyses to explore which variables (for which we treated pain score as a continuous dependent variable) to include in the ARMAX models for each cluster and retained those with significant *F* test values. Candidate variables analyzed in the ARMAX models included: age cohort, gender, BMI, pDPN duration, medical history of depression, previous use of gabapentin, history of pregabalin monotherapy, general feeling (full of energy, calm and relaxed, sad and discouraged) at Weeks 0, 1, 2, 3, 4, and 5; pain score at Weeks 0, 1, 2, 3, 4, and 5; sleep interference score at Weeks 0, 1, 2, 3, 4, 5, and 6; treatment dose at Weeks 0, 1, 2, and 3; and patient satisfaction at Weeks 0, 1, 2, 3, 4, and 5.

This matched dataset was used to derive and calibrate the ARMAX models for each of the clusters (the calibration dataset). The parameter calibration of the ARMAX models for each of the matched dataset clusters was implemented using forward and backward techniques to explore time lags and other variables to be included in each model for each cluster. A maximum likelihood method was used for the purpose of best model identification [41]. An initial validation of the ARMAX models was implemented with patients not included in the calibration dataset (i.e., patients in the Observational Study who did not match with RCT patients). A *t* test of the time series of the observed vs. predicted levels of pain was performed for validation to see if observed pain outcomes were different from those predicted with the Observational Study patients who had not matched with RCT patients.

## Results

The hierarchical cluster analysis using Ward’s minimum variance technique yielded six clusters in the Observational Study (3159 patients) with the following clustering variables: gender, age, duration of pDPN, BMI, medical history of depression, pregabalin monotherapy, prior use of gabapentin, baseline pain score, and sleep interference score at baseline. Additional file 1 shows the dendrogram and the cutoff used for identifying the six clusters. We limited the number of clusters based on the semipartial *R* ^*2*^ that measures the homogeneity of merged clusters. This value reflects decreasing homogeneity of patients in a cluster, because clusters are combined to make new clusters. As shown in the figure in Additional file 1, the cutoff at six clusters has the semipartial *R* ^*2*^ lower than 0.05, reflecting an appropriate tradeoff of low semipartial *R* ^*2*^ and not too many fragmented clusters. Ward’s minimum variance technique yielded four clusters in the RCT data alone (data not shown).

We implemented the CEM algorithm using the following four steps: 1) selected matching variables of every patient in both the Observational Study and RCT were temporarily coarsened; 2) for each cluster, all the data from the Observational Study were sorted into strata on the basis of their coarsened variables; and 3) a CEM was performed between the subgroup of patients in each cluster and all the RCT patients (more specifically: each matching variable was coarsened into substantively meaningful groups, which were then matched improving the estimation of causal effects by reducing imbalance in covariates between patients of the Observational Study belonging to a given cluster and all the RCT patients); and 4) all the patients of the Observational Study and those of the RCTs who had a coarsened exact match were included, while the other data were excluded.

There were 1204 patients in the Observational Study dataset (38%) who matched with 324 patients from the RCTs (81% of RCT patients) for a total of 1528 unique patients in the matched dataset. Table 1 highlights the similarities and differences among the clusters within this dataset. The clusters were notably distinct in many respects. For example, Cluster 1 consisted exclusively of male patients, with the highest proportion of overweight patients (67%) but low numbers of patients receiving insulin therapy (3%). In contrast, Cluster 4 was almost exclusively female (99%), with a somewhat higher proportion receiving insulin therapy (17%) and a lower incidence of overweight patients (38%). Also of note, almost all patients in Cluster 2 were on insulin (100%, the highest among all clusters), while in Cluster 3, 18% of patients were on insulin (similar to Cluster 4). However, in Cluster 2, 60% of patients received pregabalin monotherapy, compared with 0% in Cluster 3.

**Table 1:** Descriptions of the six clusters

	Cluster (*N* = 1528)^a^					
	1	2	3	4	5	6
*n*	343	306	245	195	237	202
Females (%)^b^	0.0	30.1	29.8	98.5	31.7	43.1
Age (years), mean (SD)	60.4 (8.1)	61.0 (7.7)	62.5 (7.4)	61.2 (7.6)	60.5 (9.0)	61.2 (8.5)
Age group (years), %^b^						
0–44	2.0	1.6	0.0	0.0	2.5	1.0
45–64	69.7	63.4	65.3	70.3	65.4	66.3
65–74	26.0	33.0	29.8	26.7	28.7	27.2
75+	2.3	2.0	4.9	3.1	3.4	5.5
BMI (mg/m^2^)^c^						
Mean (SD)	28.8 (3.4)	30.5 (4.4)	30.0 (4.3)	30.6 (4.8)	30.9 (5.0)	31.3 (5.8)
Normal (%)	6.4	3.3	6.5	6.7	3.0	6.4
Overweight (%)	66.8	49.4	50.2	38.0	50.2	39.1
Obese (%)	26.8	47.4	43.3	55.4	46.8	54.5
Baseline pain^b^						
Mean (SD)	6.3 (1.3)	6.5 (1.4)	6.3 (1.4)	6.5 (1.5)	6.5 (1.3)	7.0 (1.3)
Pain score (%)						
0–3	1.5	1.0	1.6	2.1	1.3	0.5
4–5	27.7	21.6	30.6	23.1	20.7	15.4
6–7	52.5	54.3	47.8	50.8	58.2	46.0
8–10	18.4	23.2	20.0	24.1	19.8	38.1
Baseline sleep interference^b^						
Mean (SD)	5.4 (2.2)	5.7 (2.2)	5.4 (2.4)	5.6 (2.3)	5.7 (2.1)	6.7 (2.2)
Sleep interference score						
0–3	22.2	16.3	22.0	19.5	14.4	10.9
4–5	26.5	30.1	26.1	25.6	30.0	15.4
6–7	33.8	28.4	29.4	33.9	38.4	34.2
8–10	17.5	25.2	22.5	21.0	17.3	39.6
Cross-correlation between sleep interference and pain^d^						
2 weeks prior based on pain in the current week (lag -2)	0.70				0.70	
1 week prior based on pain in the current week (lag -1)	0.75	0.70		0.76	0.78	
Based on pain in the current week (Lag 0)	0.85	0.81	0.77	0.85	0.87	0.81
1 week after based on pain in the current week (lag +1)	0.71	0.71		0.73	0.79	0.72
2 weeks after based on Pain in the current week (lag +2)					0.71	
Duration of pDPN (years), %^c^						
0 to ≤5	28.9	21.9	24.4	29.6	22.9	20.8
> 5 to ≤10	18.7	23.7	20.4	18.5	32.4	21.6
> 10 to ≤15	28.9	24.4	23.9	25.9	19.1	23.2
> 15 to ≤20	12.4	13.3	15.4	18.5	12.4	13.6
> 20 to ≤25	3.6	5.7	6.0	3.1	3.8	4.8
> 25	7.5	11.1	10.0	4.3	9.5	16.0
History of depression (%)^c^	0.0	0.0	0.0	0.0	18.1	100.0
Prior or current therapy (%)^c^						
Pregabalin monotherapy	100.0	59.5	0.0	100.0	58.1	40.8
Gabapentin	0.0	0.0	0.0	0.0	100.0	0.0
Insulin	3.2	100.0	18.0	16.9	78.1	57.4
Full of energy at baseline (%)						
Always	1.2	0.7	1.5	0.6	0.0	0.0
Mostly	8.1	7.9	5.0	3.7	4.8	4.8
Fairly often	11.1	6.5	11.0	11.7	8.6	8.6
Sometimes	32.8	30.5	19.9	28.4	30.5	30.5
Seldom	40.1	40.5	56.7	44.4	39.1	39.1
Never	6.6	13.3	6.0	10.5	16.2	16.2
Calm and relaxed at baseline (%)						
Always	2.4	2.5	2.0	0.0	2.9	3.2
Mostly	13.6	15.1	16.4	14.2	12.4	4.8
Fairly often	18.1	14.7	13.9	17.3	16.2	7.2
Sometimes	31.9	30.8	27.4	26.5	27.6	17.6
Seldom	32.5	30.5	37.8	35.8	32.4	50.4
Never	1.5	5.7	2.5	5.6	8.6	16.8
Sad and discouraged at baseline (%)						
Always	1.2	2.5	3.0	3.1	5.7	9.6
Mostly	15.1	14.7	13.4	16.1	12.4	42.4
Fairly often	27.1	29.0	33.3	26.5	26.7	28.8
Sometimes	32.5	29.1	29.9	23.5	27.6	7.2
Seldom	18.1	16.9	16.4	25.9	22.9	11.2
Never	6.0	7.2	3.5	3.7	4.8	0.8
Responders at 50% threshold at end (%)^c^	86.0	76.9	70.1	78.1	52.3	59.7
Daily treatment dose (mg)						
75	9.0	14.1	15.5	10.8	16.0	13.4
150	79.6	74.8	65.3	72.8	56.1	61.9
300	5.5	5.6	12.2	9.7	14.8	16.8
600	0.3	1.3	2.9	3.6	11.0	3.5
Other (30–500 mg, excluding doses above)	5.5	4.3	4.1	3.1	2.1	4.5

*Abbreviations: SD* standard deviation, *BMI* body mass index, *pDPN* painful diabetic peripheral neuropathy

^a^Each cluster analysis was derived from the Ward’s minimum variance technique that grouped patients in such a way that patients in the same group (called a cluster) were more similar to each other than to those in other clusters

^b^Variables used to create the clusters

^c^Other variables not used for creating the clusters

^d^Only cross-correlations ≥0.70 are shown

Of the 324 patients who matched, 17% of RCT patients matched to one cluster, 23% to two clusters, and 60% to three or more clusters. The reduction in the imbalance scores for the clusters after adding in the RCT patients (ranging from 6 to 63% depending on the cluster) suggests that the process reduced the bias of covariates notably in five of the six clusters with only Cluster 1 retaining a relatively higher imbalance of covariates (see Table 2).

**Table 2:** CEM results

Cluster	Patients by cluster, *n*			Global imbalance^a^		Reduction in global imbalance^a^ After CEM (%)
	Observational Study Alone		Observational Study + RCT in matched dataset After CEM, *n*			
	Before CEM	After CEM		Before CEM	After CEM	
1	696	332	343	0.72	0.68	6
2	777	279	306	0.70	0.26	63
3	542	201	245	0.72	0.27	63
4	556	162	195	0.84	0.33	61
5	287	105	237	0.74	0.30	59
6	301	125	202	0.71	0.30	58
Total	3159	1204^b^	1528			

*Abbreviations: CEM* coarsened exact matching, *RCT* randomized controlled trial

^a^The degree of imbalance represents level of bias in the covariates’ distributions for a given sample. According to Iacus et al. (2008) [56], *‘the key goal of matching is to prune observations from the data so that the remaining data have better balance between the control and the treated groups’* (e.g., the observational study dataset of each cluster and RCT data). *‘Exactly balanced data [*i.e.*, global imbalance score = 0] means that controlling further for X is unnecessary (since it is unrelated to the treatment variable), and so a simple difference in means on matched data can estimate the causal effect; approximately balanced data requires controlling for X with a model (*e.g.*, the same model that would have been used without matching), but the only inferences necessary are those relatively close to the data, leading to less model dependence and reduced statistical bias than without matching.’* (See: 1) Imbens GW, Rubin DB. Causal inference in statistics, social, and biomedical sciences. Cambridge, UK: Cambridge University Press; 2015 [57]. 2) King G, Lucas C, Nielsen R. The balance-sample size frontier in matching methods for causal inference. Am J Poli Sci. doi:10.1111/ajps.12272 [58]. 3) Stuart EA. Matching methods for causal inference: a review and a look forward. Stat Sci. 2010;25:1–21 [59].) Therefore, in our case, an imbalance of 0 means that the empirical distribution of the covariates of the Observational Study dataset in a given cluster is equivalent to in the RCT data; an imbalance of 1 means that the empirical distribution is completely different

^b^One hundred sixty-two of them were excluded from the ARMAX model calibration because they lacked pain and sleep interference data for the full six weeks; hence there were 1042 observational study patients in the calibration dataset for developing the ARMAX models

**Fig. 1:**
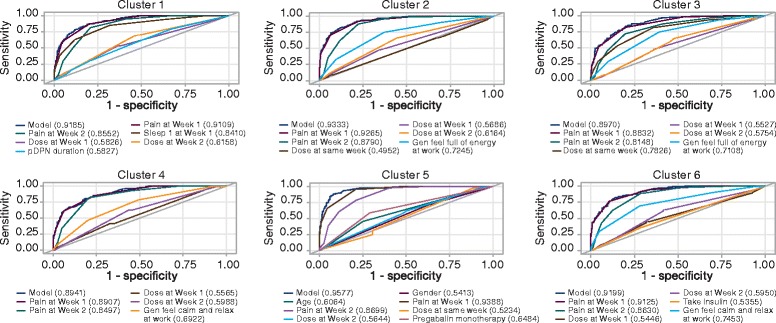
ARMAX model ROC curves for 50% responder levels the six clusters^a^. ^a^Attaining the responder level of 50% is the dependent variable for these models in contrast to pain score, which is the dependent variable in the models in Table 3. *ROC* receiver operating characteristic

The final ARMAX models estimating weekly pain scores for the matched data (calibration dataset) are shown in Table 3. All the models performed well, with *R* ^*2*^ ranging from 0.85 to 0.91 and root mean square errors ranging from 0.53 to 0.57. We also generated receiver operating characteristic curves for whether or not the patient achieved responder status with ‘pain responder level’ defined as: (pain score at baseline – pain score (t))/pain score at baseline at the 50% threshold. These results are shown in Fig. 1. The most influential variables were those associated with time-lagged relationships: 1) pain (at one and two weeks prior to predicted pain at a given week); 2) dose (at one and two weeks prior); and 3) sleep interference (at one week prior). The following were influential in one or several clusters: feeling full of energy in the week before, feeling calm and relaxed in the week before, insulin use (yes or no), age group, gender, pregabalin (monotherapy or combination therapy), and dose given.

**Table 3:** ARMAX model input variables and regression coefficients by cluster for the calibration dataset

ARMAX model input variables	Final ARMAX output regression coefficients, by cluster^a^					
	1	2	3	4	5	6
y-intercepts for regression models, not variables	−0.0409	−0.1447	−0.0604	−0.0826	0.0789	−0.2732
Age cohort (×10)	–	–	–	–	0.0465	–
Gender (×9)	–	–	–	–	−0.0496	–
Pregabalin monotherapy (×8)	–	–	–	–	−0.1107	–
pDPN duration (years) (×11)	0.0179	–	–	–	–	–
Insulin use (×7)	–	–	–	–	–	0.0276
Pain score (t-1)^b^ (×1)	0.7180	0.8749	0.7865	0.8341	0.9011	0.8919
Pain score (t-2)^c^ (×2)	0.0436	0.0196	0.0164	0.0451	0.0107	−0.0103
Sleep interference score (t-1)^b^ (×3)	0.0949	–	0.0374	–	–	–
Dose (t)^d^ (×4)	–	−0.0006	–	–	−0.0011	–
Dose (t-1)^b^ (×5)	−0.0012	−0.0007	−0.0009	−0.0012	–	−0.0004
Dose (t-2)^c^ (×6)	0.0015	0.0015	0.0012	0.0017	0.0012	0.0007
General feeling: full of energy (t-1)^b^ (×13)	–	0.0250	0.0425	–	–	–
General feeling: calm and relaxed (t-1)^b^ (×12)	–	–	–	−0.0109	–	0.0655
Model performance measures applied	Performance, by cluster					
	1	2	3	4	5	6
Likelihood ratio *P* value	<0.0001	<0.0001	<0.0001	<0.0001	<0.0001	<0.0001
*R* ^*2*^	0.86	0.89	0.85	0.87	0.91	0.89
Root mean square error	0.54	0.55	0.55	0.53	0.53	0.57
Observed vs. estimated responder level (Student’s *t* test *P* value)^e^	0.95	1.00	0.95	0.97	0.92	0.96

*Abbreviations: ARMAX* autoregressive moving average model, *pDPN* painful diabetic peripheral neuropathy

^a^The first number in each column is the regression intercept value. Blank spaces in columns indicate that the associated row variable was not a predictor in the final model for that cluster

^b^(t-1) indicates one week before prediction

^c^(t-2) indicates two weeks before prediction

^d^(t) indicates the same week of the prediction

Given the time series of pain scores, ARMAX is essentially a linear regression model for understanding future values of pain scores in the series. The ARMAX model inputs were assigned unique variable names, x1x13, and are represented in the cluster-specific ARMAX equations below

Equations for the ARMAX models (where ‘y’ is pain score, treated as a continuous variable)

CLUSTER 1: y = −0.0409 + 0.7180 × 1 + 0.0436 × 2 + 0.0949 × 3–0.0012 × 5 + 0.0015 × 6 + 0.0179 × 11

CLUSTER 2: y = −0.1447 + 0.8749 × 1 + 0.0196 × 2–0.0006 × 4–0.0007 × 5 + 0.0015 × 6 + 0.0250 × 13

CLUSTER 3: y = −0.0604 + 0.7865 × 1 + 0.0164 × 2 + 0.0374 × 3–0.0009 × 5 + 0.0012 × 6 + 0.0425 × 13

CLUSTER 4: y = −0.0826 + 0.8341 × 1 + 0.0451 × 2–0.0012 × 5 + 0.0017 × 6–0.0109 × 12

CLUSTER 5: y = 0.0789 + 0.9011 × 1 + 0.0107 × 2–0.0011 × 4 + 0.0012 × 6–0.1107 × 8–0.0496 × 9 + 0.0465 × 10

CLUSTER 6: y = −0.2732 + 0.8919 × 1–0.0103 × 2–0.0004 × 5 + 0.0007 × 6 + 0.0276 × 7 + 0.0655 × 12

^e^The ARMAXs estimate pain score, but we also want to be able to identify whether that patient is a responder at different thresholds (e.g., 50% reduction in pain or 30% reduction in pain). Hence, we sought to confirm estimation of responder level based on the ARMAXs for pain score

The results of how well these ARMAX models also predicted responders in Observational Study patients who did not match with RCT patients are summarized in Table 4. We used two-sample *t* tests in this validation dataset (*n* = 1955) to compare observed pain scores in the validation dataset with those predicted using the ARMAX models derived based on the calibration dataset. The left panels of Additional file 2 show histograms of the percent distribution of patients by pain score (0–10) both for observed and ARMAX-predicted findings for each of the clusters. The right panels show similar plots for all clusters, but for patient distribution by percent change in response (10% increments). All models showed *P* values indicating no significant differences (*P* values ranging from 0.26–0.83) for Student’s two-sample *t* tests comparing the observed and predicted outcomes for the various pain scores and percent changes in response.

**Table 4:** ARMAX model predictive capability for pain and responder status in the validation dataset

Clusters^a^	Observed vs. estimated, *P* value			
	Pain level for responders		Responder status achieved	
	At 50% threshold	At 30% threshold	At 50% threshold	At 30% threshold
1	0.52	0.31	0.50	0.20
2	0.78	0.16	0.65	0.11
3	0.29	0.22	0.26	0.14
4	0.71	0.32	0.83	0.28
5	0.66	0.42	0.73	0.25
6	0.76	0.30	0.79	0.25

*Abbreviations*: *ARMAX* autoregressive moving average model, *RCT* randomized controlled trial

^a^Observational study dataset of patients not matched with RCT patients

The results of how well the maximum likelihood regressions performed before and after CEM matching are shown in Additional file 3. The log likelihoods of the regressions for each of the cluster were significantly better based on the likelihood ratio chi-square test (*P* < 0.0001). The improvement in the predictive capability of all the clusters after matching with the RCT dataset also is confirmed by the substantive increase of the log-likelihood value after CEM (i.e., higher log-likelihood values mean higher explanatory capability of the matching variables on pain score at baseline). The significance of this improvement in the log likelihood score after matching is evidenced by the outcomes of a chi-square test between the log likelihood of the logit models of pain at baseline in relation to the matching variables (i.e., sex, age, BMI, sleep interference at baseline) before and after application of CEM.

## Discussion

These findings highlight the complexity of the characteristics that comprise responders to pregabalin. Table 1 showed the similarities and differences among a number of variables across the clusters; yet these variables combined differently to predict response in the different clusters as seen in the ARMAX results in Table 3 and Fig. 1. The parameters in the ARMAX models reinforced the reciprocal influences between pain and sleep interference [42] and dose in previous weeks [43, 44]. They also showed the relevance of selected psychosocial variables (e.g., calm and relaxed, full of energy) for certain subgroups of patients, but not others, as has been shown in other studies [8, 17]. Other variables such as age, gender, pDPN duration, and pregabalin monotherapy were the only significant predictors in one of the responder subgroups, although such characteristics are often used as a basis for subgroup analyses in clinical studies of pain [11, 17, 45–48].

The value of creating clusters of patients with similar characteristics in order to better predict response was intuitively evident. One possible explanation for the usefulness of our combination of clustering and matching techniques for predicting weekly pain scores over time is the inclusion of adequate time series dynamics for pain scores and sleep interference blended with other patient characteristics. The robustness of the predictions was confirmed by the strong performance of the ARMAX models in the validation dataset summarized in Table 4 because these Observational Study patients had not matched with any RCT patients and were consequently different. The strong predictive capability of the ARMAX models in each cluster suggests that it is feasible to predict the magnitude of the response to pregabalin when useful subgroups (clusters) of patients are created first. Moreover, because we predict weekly pain scores with the ARMAXs, we are not limited to a specific threshold for percent change in pain response with the models developed for each of these clusters (see Additional file 2). Finally, using Ward’s minimum variance as a clustering technique offered a reasonable tradeoff between the number of observations in each cluster and the homogeneity of the patients in a specific cluster as measured by traditional cluster analyses performance measures (see Additional file 1).

### Integration of RCT and Observational Study data

A related justification for why the ARMAX models were able to predict pain responses was because of the use of RCT data to reduce covariate bias in the Observational Study dataset after it was separated into patient clusters. The reduction in the imbalance scores for the clusters after adding in the RCT patients (ranging from 6 to 63% depending on the cluster, as shown in Table 2) suggests that the process reduced the bias of covariates notably in five of the six clusters, with only Cluster 1 retaining a relatively higher imbalance of covariates. This interpretation also is confirmed by both the increase and the statistical significance of the log-likelihood values after CEM of the logit model of pain score (see Additional file 3).

The relevance and importance of both RCT and Observational Study data in their utility for predicting patient outcomes was affirmed. The cluster analysis enabled matching of 81% of the RCT patients, suggesting notable overlap of most RCT patients with Observational Study patients, despite the geographic differences in the location of the patients in these studies. Since over 60% of the RCT patients matched to three or more clusters and only 17% matched to one cluster, we effectively weighted the randomized patients by allowing the multiple matches. These findings also supported starting with the Observational Study data and matching RCT patients to it, because a greater number of RCT patients had multiple matches than if we had started with the RCT and matched Observational Study patients (2154 vs 1823 total, non-unique patients, an 18.4% increase). The results also confirm that the expected broader spectrum of patients does exist in the Observational Study because only 38% of the Observational Study patients matched with these RCT patients. However, these other 62% of Observational Study patients’ responses could be predicted with our cluster-based ARMAX models, suggesting that, while they are different on matching variables, the predictive relationships for outcomes are present. One possible explanation for this finding is the reduction of covariate bias that was achieved with CEM. The differences in the imbalance of covariates, however, did not fully align with predictive capabilities. While performance in all clusters improved after CEM, the ARMAX models performed better for four of the five clusters with lower global imbalances (Clusters 2, 4, 5, 6). The exception was Cluster 3 that, although having one of the lower global imbalance scores, had a predictive capability in the validation dataset that was not as good as the other clusters’ ARMAXs.

We pursued this overall methodological approach in order to benefit from the advantages of both RCTs and observational studies and have now demonstrated a proof of concept regarding a predictive analytical approach to the integration of observational study and RCT patient data (that offers a step toward the ultimate goal of precision medicine [14]. We rely on evidence from randomized clinical research and observational real-world investigations to make medication treatment choices. These choices require clinicians to blend evidence derived from research focused on internal validity to assess cause and effect together with research focused on external validity to evaluate relevance to a specific treatment decision. Concato et al. (2000) compared RCTs and observational studies on the same topic (99 studies in five topics) and found that well-designed observational studies do not systematically over/underestimate the magnitude of the effects of treatment as compared with those in RCTS on the same topic, and that each are valuable in delivering evidence helpful to patient care [49]. Benson et al. (2000) analyzed 136 reports about 19 diverse treatments and concluded similarly that there is little evidence that estimates of treatment effects in observational studies are either consistently larger than or qualitatively different from those obtained in RCTs [50]. Given the importance of both types of studies, efforts to directly link them by reducing potential covariate biases in observational studies can improve treatment choices and patient outcomes. Others have used CEM in other disease areas to reduce multivariate imbalance and thereby improve regression model estimates [51–54].

### Limitations

One limitation is that the ARMAX models are based on only three RCTs (combined *N* = 398) and one large Observational Study (*N* = 3159 patients) that were evaluated for this initial proof of concept. As with any study, bias from omitted covariates cannot be eliminated. Based on our encouraging findings, we have launched ongoing work to expand the datasets. Another possible consideration is that we might be able to predict outcomes even better if the differences between clusters produced with Ward’s minimum variance technique were more distinct from one another. Ongoing work with other clustering and machine learning techniques will enable us to see the relative importance of the specific clustering methods for our predictions.

Another limitation is that we decided up front to focus on predicting responders who completed the studies (and thus tolerated side effects). Those who experienced adverse events and discontinued the studies were excluded. This was a logical starting place for the proof of concept; subsequent analyses can focus on identifying those who would likely discontinue for safety or tolerability reasons as well as incorporating statistical techniques for handling missing data.

Another limitation is the extent to which we may currently extend and apply implications of our findings to novel patients at clinical presentation. Additional work is ongoing to extend the predictive capabilities of ARMAX models using agent-based modeling and simulation techniques [55]. This work has focused on predicting response to pregabalin using baseline values of the patient variables in the ARMAX models in order to assign novel patients to particular clusters. The work also includes predicting outcomes based on changes after 1 week or several weeks of treatment and dose adjustments to discern how to implement prediction in a practical way in a clinical setting with an accessible user interface.

These findings are specific to patients with pDPN, which is another limitation. Other clinical circumstances may require less or more complex approaches to enable prediction. While our results are specific to patients with pDPN, they suggest that these techniques should be explored with larger datasets of both RCTs and observational studies, and with different clustering and matching techniques, in order to better understand when clustering and matching can help us predict medication responders more effectively.

## Conclusions

The six clusters identified were distinct, but with many similarities and specific differences. Though often used as a basis for prospective subgroup analyses in clinical studies in neuropathic pain, exogenous variables such as age, gender, pDPN duration, and pregabalin as monotherapy or as concomitant therapy  were rarely predictive in and of themselves. It was their different combinations in concert with reciprocal influences between pain and sleep interference that predicted response. These relationships help explain why it is challenging to predict consistently the right treatment for the right patient. The ARMAX models also highlighted the importance of pregabalin dose in the prior weeks and its role in conjunction with these variables in predicting responders.

The other important consideration in effective prediction of responders that was seen in these analyses related to the improved performance of the models based on blending of randomized and observational data to reduce the covariate biases in observational studies. The CEM technique enabled use of the advantages of randomization to enrich the patient data collected to identify responders in a more real world setting by affording reductions in the inherent biases that occur from covariates in observational data. The use of combined data from a large German Observational Study and three pivotal North American RCTs to generate these clusters suggests that implementation of time series–based multivariable models at the patient subgroup level (clusters) offers a way to put similar patients together. The finding that RCT-derived data could be used to develop better models that predict patient outcomes in a broader spectrum of Observational Study patients with different characteristics than those in the RCTs supports the potential practical aspects of this approach, pending confirmation with more studies and applications beyond pDPN. Possible other advanced modeling and machine learning techniques also could be useful in these efforts because of their ability effectively to handle complex relationships among variables changing over time.

## Additional files

Additional file 1: Cluster analysis performance. (PDF 335 kb)

Additional file 2: Results of validation of the ARMAX models of the six clusters. (PDF 824 kb)

Additional file 3: Log likelihood of multilogit pain regression on cluster variables before and after CEM. (PDF 192 kb)

## Abbreviations

ARMAX: AutoRegressive Moving Average models with eXogenous inputs
BMI: Body mass index
CEM: Coarsened Exact Matching
COMBO-DN: COmbination versus Monotherapy of pregaBalin and dulOxetine in Diabetic Neuropathy Study
EM: Exact matching
HbA1c: glycated hemoglobin
NeuPSIG: Special Interest Group on Neuropathic Pain
pDPN: Painful diabetic peripheral neuropathy
PSM: Propensity score matching
RCT: Randomized controlled trial
