# Supplementary material for: Integrating data from randomized controlled trials and observational studies to predict the response to pregabalin in patients with painful diabetic peripheral neuropathy
Source: BMC Med Res Methodol. 2017 Jul 20;17:113. doi: 10.1186/s12874-017-0389-2 (PMC5520324; doi:10.1186/s12874-017-0389-2)
Supplement: Supplementary file 1 — Cluster analysis performance. (PDF 335 kb) [file 12874_2017_389_MOESM1_ESM.pdf]

Additional file 1 Cluster analysis performance. Ward's minimum variance cluster analysis dendrogram.[a]

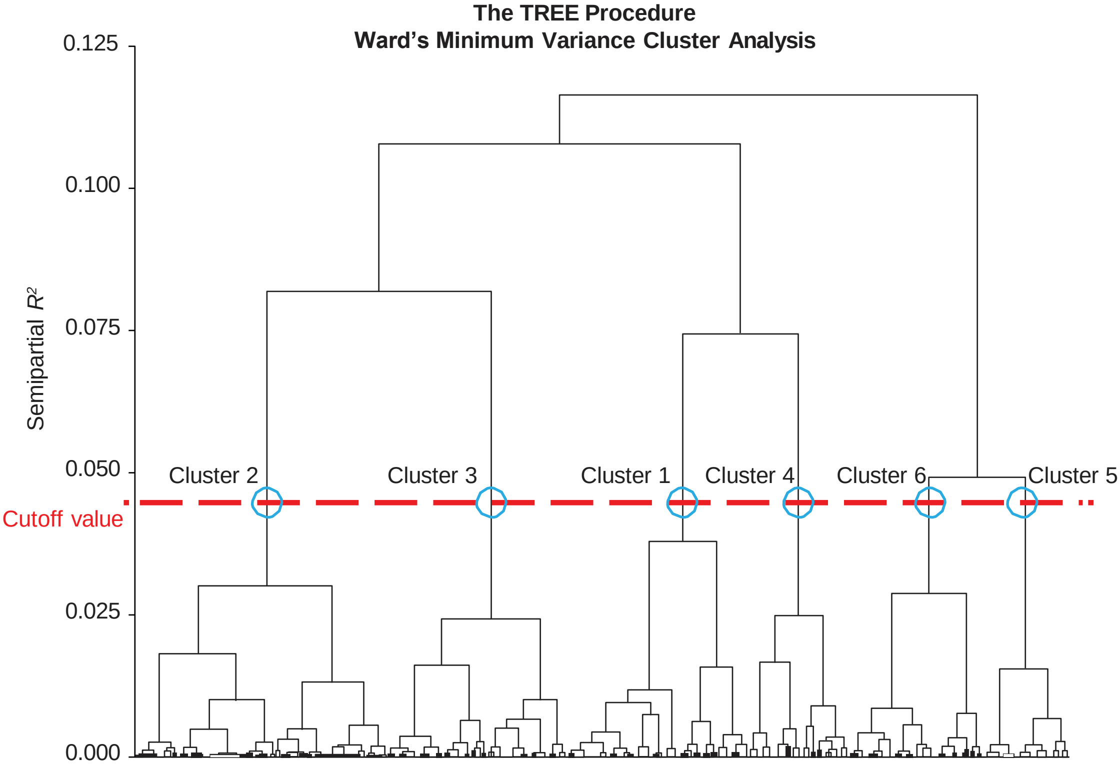

[a]A dendrogram plot consists of many *U*-shaped lines that connect data points in a hierarchical tree. The height of each *U* represents the distance between the two data points being connected

Pseudo-*F* statistic plot interpretation (PSF); pseudo-T^2 statistic plot interpretation (PST). As indicated in the figure below, six is the number of clusters that either is associated to both the peak of cubic clustering criterion (CCC) value and the first greater change in the PST value. It also is very close to the number of clusters corresponding to the PSF peak value.

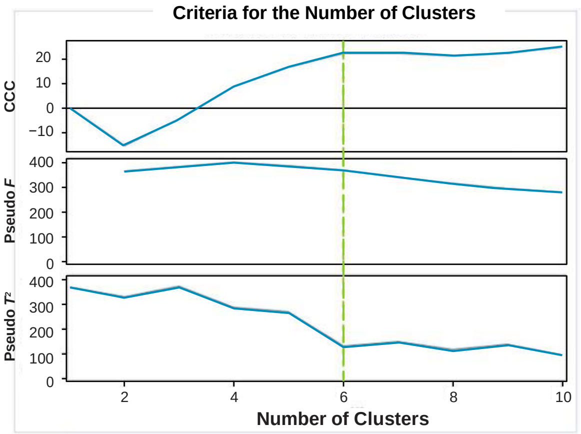

Estimating the number of clusters

Three methods that can be used to estimate the number of clusters are:

1. CCC method: the estimated number of clusters occurs at the start of a peak on the graph. There may be more than one peak per plot.
2. Pseudo *F*: estimated number of clusters occurs at the start of peaks on the graph. There may be more than one peak per plot.
3. T^2: the graph is read right to left. The estimated number of clusters occurs at the start of a peak. There may be more than one peak per plot.

Cluster history

No. of clusters	Clusters joined		Frequency	Semipartial R^2	R^2	Approximate expected R^2	CCC	Pseudo *F* statistic	Pseudo T^2	Tie
10	CL23	CL12	469	0.0157	0.448	0.407	25.0	284	94.5	
9	CL16	CL25	556	0.0169	0.431	0.393	22.4	298	135.0	
8	CL19	CL20	542	0.0176	0.413	0.375	21.4	317	116.0	
7	CL29	CL13	696	0.0178	0.395	0.354	22.7	344	147.0	
6	CL10	CL18	777	0.0254	0.370	0.327	22.3	370	130.0	
5	CL7	CL9	1,252	0.0403	0.330	0.296	16.5	388	267.0	
4	CL5	CL8	1,794	0.0524	0.277	0.259	8.72	404	286.0	
3	CL4	CL6	2,571	0.0804	0.197	0.207	–4.7	387	371.0	
2	CL11	CL14	588	0.0926	0.104	0.136	–16.0	368	328.0	
1	CL3	CL2	3,159	0.1043	0.000	0.000	0.0	–	368.0	
