# Supplementary material for: Integrating data from randomized controlled trials and observational studies to predict the response to pregabalin in patients with painful diabetic peripheral neuropathy
Source: BMC Med Res Methodol. 2017 Jul 20;17:113. doi: 10.1186/s12874-017-0389-2 (PMC5520324; doi:10.1186/s12874-017-0389-2)
Supplement: Supplementary file 2 — Results of validation of the ARMAX models of the six clusters. (PDF 824 kb) [file 12874_2017_389_MOESM2_ESM.pdf]

Additional file 2 Results of validation of the ARMAX models of the six clusters

Distribution of Observed Pain vs. Estimated Pain Score (validation dataset cl. 1)

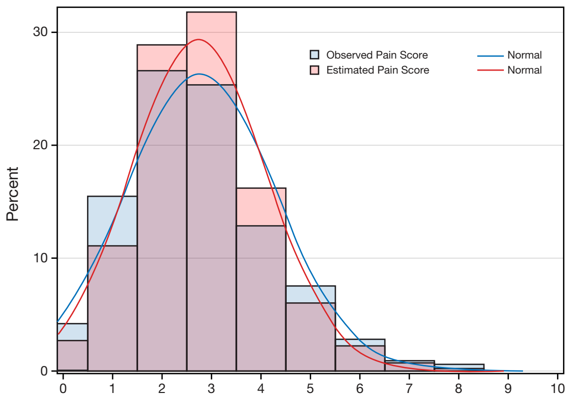

Distribution of Observed Response vs. Estimated Response (validation dataset cl. 1)

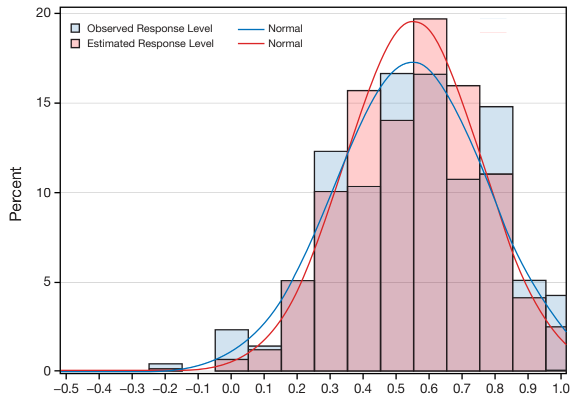

Cluster 1: validation based on 294 patients (1,470 weekly observations, 5 observations per patient) of Observational Study dataset patients not matched with RCTs (*P* value of observed vs. predicted pain score = 0.52; *P* value observed vs. predicted percent change in response = 0.50)

Distribution of Observed Pain vs. Estimated Pain Score (validation dataset cl. 2)

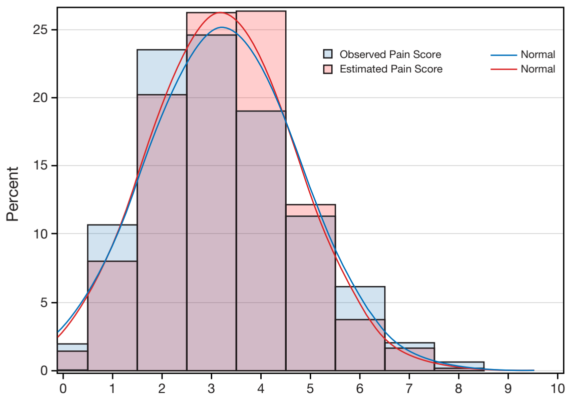

Distribution of Observed Response vs. Estimated Response (validation dataset cl. 2)

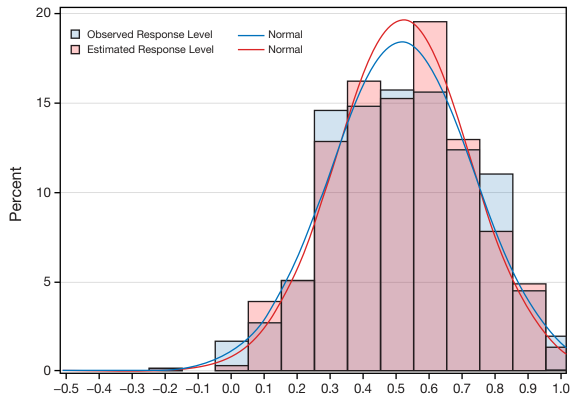

Cluster 2: validation based on 407 patients (2,035 weekly observations, 5 observations per patient) of 1,061 Observational Study dataset patients not matched with RCTs (*P* value of observed vs. estimated pain score = 0.78; *P* value observed vs. estimated pain responder level = 0.65)

Distribution of Observed Pain vs. Estimated Pain Score (validation dataset cl. 3)

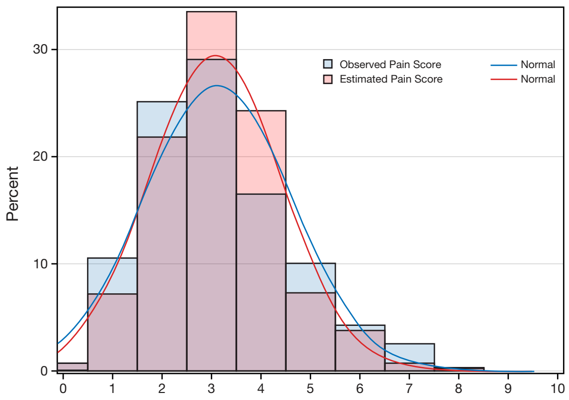

Distribution of Observed Response vs. Estimated Response (validation dataset cl. 3)

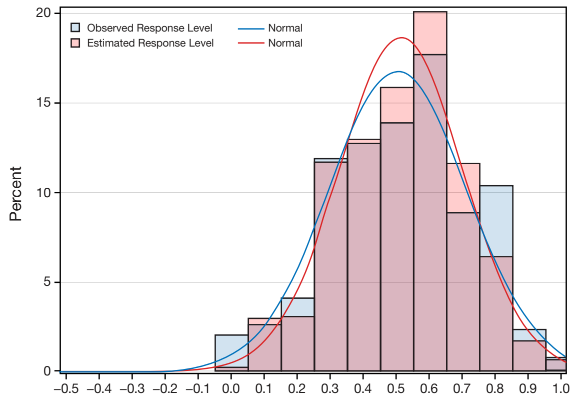

Cluster 3: validation based on 274 patients (1.370 weekly observations, 5 observations per patient) of 1,061 Observational Study dataset patients not matched with RCTs (*P* value of observed vs. estimated pain score = 0.29; *P* value observed vs. estimated pain responder level = 0.26)

Distribution of Observed Pain vs. Estimated Pain Score (validation dataset cl. 4)

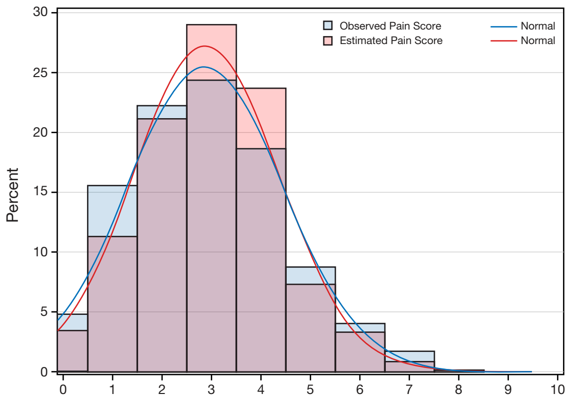

Distribution of Observed Response vs. Estimated Response (validation dataset cl. 4)

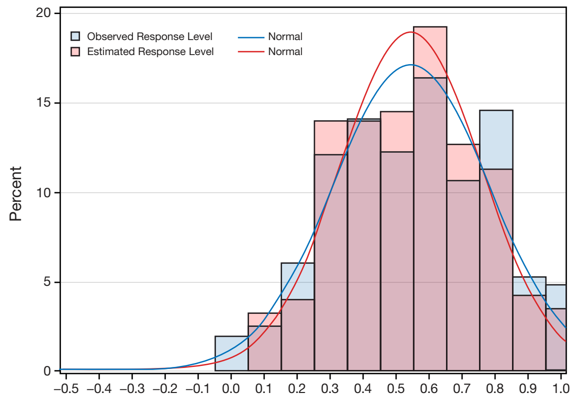

Cluster 4: validation based on 321 patients (1,605 weekly observations, 5 observations per patient) of 1,061 Observational Study dataset patients not matched with RCTs (*P* value of observed vs. estimated pain score = 0.71; *P* value observed vs. estimated pain responder level = 0.83)

Distribution of Observed Pain vs. Estimated Pain Score (validation dataset cl. 5)

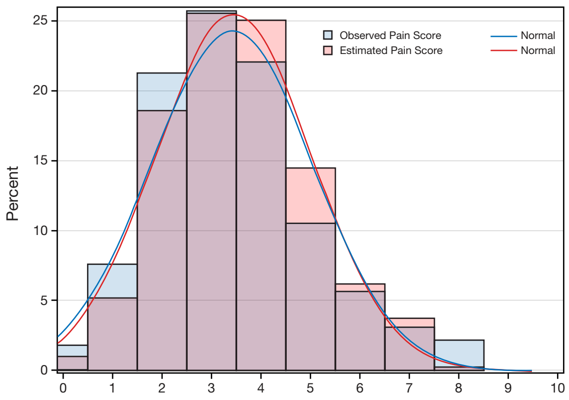

Distribution of Observed Response vs. Estimated Response (validation dataset cl. 5)

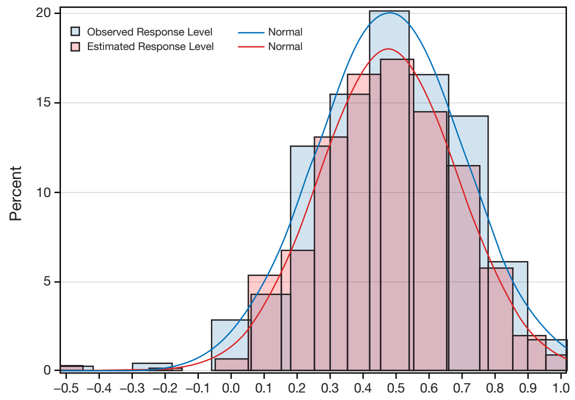

Cluster 5: validation based on 155 patients (775 weekly observations, 5 observations per patient) of 1,061 Observational Study dataset patients not matched with RCTs (*P* value of observed vs. estimated pain score = 0.66; *P* value observed vs. estimated pain responder level = 0.73)

Distribution of Observed Pain vs. Estimated Pain Score (validation dataset cl. 6)

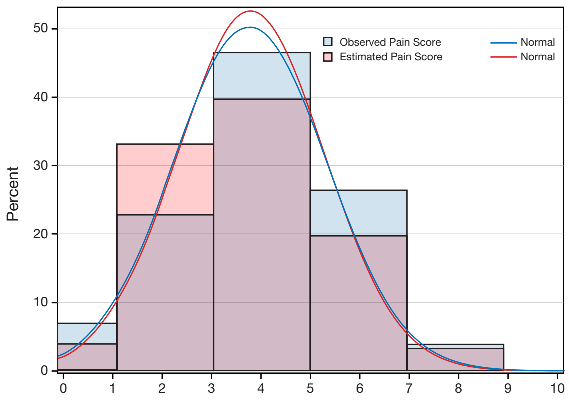

Distribution of Observed Response vs. Estimated Response (validation dataset cl. 6)

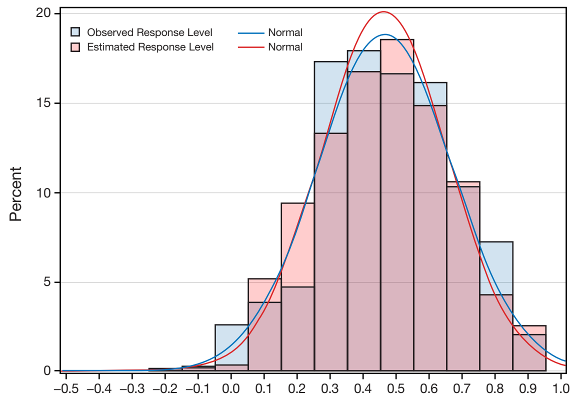

Cluster 6: validation based on 149 patients (745 weekly observations, 5 observations per patient) of 1,061 Observational Study dataset patients not matched with RCTs (*P* value of observed vs. estimated pain score = 0.76; *P* value observed vs. estimated pain responder level = 0.79)
ARMAX autoregressive moving average model, RCT randomized controlled trial
