# Supplementary material for: Integrating data from randomized controlled trials and observational studies to predict the response to pregabalin in patients with painful diabetic peripheral neuropathy
Source: BMC Med Res Methodol. 2017 Jul 20;17:113. doi: 10.1186/s12874-017-0389-2 (PMC5520324; doi:10.1186/s12874-017-0389-2)
Supplement: Supplementary file 3 — Log likelihood of multilogit pain regression on cluster variables before and after CEM. (PDF 192 kb) [file 12874_2017_389_MOESM3_ESM.pdf]

Additional file 3 Log likelihood of multilogit pain regression on cluster variables before and after CEM

Cluster	Log likelihood[a] before CEM (LM1)	Log likelihood after CEM (LM2)	Chi-square test[b]	*P* value (chi-square test)	(LM1 – LM2)/LM1[c]
1	–1826.8	–669.6	2314.4	<0.0001	63
2	–2211.7	–821.9	2779.5	<0.0001	63
3	–1563.1	–473.4	2179.4	<0.0001	70
4	–1595.2	–287.8	2614.8	<0.0001	82
5	–1152.6	–237.5	1830.3	<0.0001	79
6	–1126.8	–335.6	1582.4	<0.0001	70

Abbreviations: CEM coarsened exact matching

[a]Log likelihood: The goal of maximum likelihood regression is to determine optimal values of the estimated coefficients. Log likelihood values cannot be used alone as an index of fit because they are a function of sample size but can be used to compare the fit of different coefficients.

[b]The likelihood ratio chi-square tests between the two log likelihoods (before and after CEM)

[c]Improvement of the log likelihood after CEM. In our case, log likelihood is the natural logarithm of the likelihood function of the pain score at Baseline in relation to the matching variable. It is a monotonically increasing function that describes the explanatory capability of the matching variables on pain score at Baseline. The desired goal is to maximize the log likelihood. Higher log-likelihood values mean higher explanatory capabilities of the matching variables on pain score at Baseline.
